# Heredity of Syphilis

**Published:** 1894-01

**Authors:** 


HEREDITY OF SYPHILIS.—Bergh states that, while it is generally acknowledged that syphilis may be inherited as well from the father as from the mother, it has been doubted that the child may inherit syphilis from the father while the mother remains in perfect health without syplilitic taint. Bergh has observed a case which appears to prove the possibility of this condition.

A young prostitute who never had suffered from syphilis was delivered of a female child, who, three weeks after birth, presented evident symptoms of hereditary sypilis (erythema syphil., ulcers in the anal region, coryza). The symptoms disappeared after treatment with calomel. More than six months after the birth of the child the mother came under treatment for recently-acquired syphilis, with indurated cancre, enlargement of the inguinal glands, etc. The child had been reared under circumstances in which it was not likely to acquire the disease, the symptoms being those of inherited, not acquired syphilis; and the fact that six months later the mother was taken ill with the primary symptoms seems to prove that she must have bean healthy at the time of childbirth.—*Hospitalstidende*, 1893, *p.* 697.—*Universal Medical Journal.*

